# Cellular correlates of selfish spermatogonial selection†

**DOI:** 10.1111/andr.12185

**Published:** 2016-04-26

**Authors:** G. J. Maher, E. Rajpert‐De Meyts, A. Goriely, A. O. M. Wilkie

**Affiliations:** ^1^ Weatherall Institute of Molecular MedicineUniversity of OxfordOxfordUK; ^2^ Department of Growth and ReproductionCopenhagen University Hospital (Rigshospitalet)CopenhagenDenmark

The mechanisms leading to increased genetic risks for offspring of older fathers have received increased scrutiny with improvements in genomics technologies. For example, there is now convincing genetic evidence that, aside from the approximately linear increase in paternally originating point mutations with age (Kong *et al*., 2012; Rahbari *et al*., 2016), there is a steeper age rise for a small subset of mutations conferring gain‐of‐function properties. This is best documented for mutations in the *FGFR2*,* FGFR3*,* HRAS, PTPN11* and *RET* genes, all of which act in growth factor receptor‐RAS signalling, a pathway known to be active in spermatogonia and that is commonly dysregulated in cancer (reviewed by Goriely & Wilkie, 2012). The mutations are proposed to provide a selective advantage for proliferation and/or survival to mutant spermatogonia (Goriely *et al*. 2003), leading to clonal expansion over time: a process that we have termed *selfish spermatogonial selection*.

In an attempt to search for the cellular correlates of this process, we developed immunohistochemical methods to detect unusual groups of cells within the seminiferous tubules of formalin‐fixed, paraffin embedded (FFPE) human testes (Lim *et al*., 2012). Recently Pohl *et al*. (2016) have contributed a Short Communication that has questioned the interpretation of our previous work. Briefly, when they examined Bouin's‐fixed testis biopsies from men of three different age groups (~29, ~48 and ~72 years), these authors found no change in the prevalence of cellular clusters, whereas an increase with age might be expected if they were caused by selfish selection. Moreover, Pohl *et al*. proposed and illustrated how the appearance of cellular clusters may arise artefactually, especially from oblique or tangential sections of seminiferous tubules as they make sharp U‐turns. Whilst we agree (and emphasized in Lim *et al*., 2012) that great care must be taken to avoid artefactual interpretation of testis sections, we now have additional genetic data, discussed below, which demonstrate that we have directly identified the cellular correlates of selfish spermatogonial selection.

In the Discussion of our original paper (Lim *et al*., 2012), we made a clear distinction [which Pohl *et al*. (2016) did not appear to take full account of] between two immunohistochemical phenomena visible in testis sections: (i) putative ‘microclones’, defined as small clusters of MAGEA4‐expressing spermatogonial cells located at the periphery or within the lumen of tubular cross‐sections of seminiferous tubules, and (ii) ‘immunopositive tubules’, characterized by abnormal staining of the entire circumference of the tubular cross‐sections. The latter were readily visible at low magnification, often observed in groups, and displayed an overall darker staining when compared to their neighbours. In discussing these two phenomena, we were explicit that the size and frequency characteristics of the ‘microclones’ (93% of them comprised fewer than 200 cells) ‘*do not match the expectation of mutational clones previously described by DNA studies*’ (Lim *et al*., 2012). In our view, all the work presented by Pohl *et al*. (2016) concerns this ‘microclone’ (or, in their words, ‘microcluster’) appearance, the significance of which remains to be determined. We agree that artefactual explanations for microclusters are possible, especially when only MAGEA4 staining is employed, as was the case in Pohl *et al*. (2016) – note that in addition to spermatogonia, weaker MAGEA4 expression can also be detected in spermatocytes. In Lim *et al*. (2012), we utilized serial sections and employed up to seven different antibodies to characterize adjacent testicular sections, and documented that in many instances, contiguous cells in a cluster could be independently identified, using different spermatogonial markers. Whatever the significance of these microclones/microclusters may be, in our opinion, the approach taken by Pohl *et al*. (2016) did not address the primary biological question concerning whether selfish clones can be identified.

**Figure 1:**
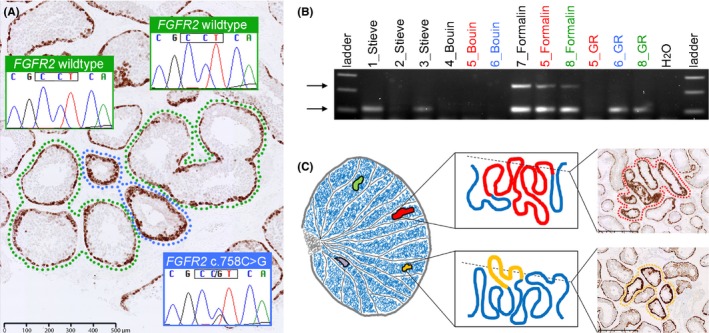
Selfish spermatogonial selection at the cellular level: confirmation, investigation and modelling. (A) This MAGEA4‐stained section of formalin‐fixed testis, removed from a 39‐year‐old man because of an inguinal hernia, shows two adjacent tubular cross‐sections (blue surround) displaying consistently increased immunopositivity at the tubular periphery, compared to the normal appearance of the neighbouring tubular cross‐sections (green or no surround). Adjacent sections stained with FGFR3 and phospho‐AKT also exhibited increased immunopositivity (not shown), ruling out the possibility of a staining artefact. Genetic analysis of microdissected tubules showed that the *FGFR2* c.758C>G (p.P253R) substitution, which causes Apert syndrome in the germline and occurs in endometrial cancer as a somatic mutation, was present in the immunopositive tubules, but not in the adjacent normally stained tubules, confirming the correlation between the immunopositive appearance and the presence of a selfish pathogenic mutation. Scale bar: 500 μm. Full details are provided in Fig. S7 of Maher *et al*. (2016). (B) Commonly used fixatives for testicular biopsies result in DNA degradation. DNA extraction was performed on 12 testicular biopsies processed in four different fixatives for similar durations. Multiple biopsies from three testes (5, 6, and 8, colour‐coded red, blue and green respectively) were immersed in different fixatives, enabling direct comparison of their impact on DNA degradation. Here, 35 cycles of PCR were performed, using 20 ng of DNA as template, with multiplexed *GAPDH* primers and cycling conditions as outlined by Agilent Technologies FFPE‐Derived DNA Quality Assessment guide (G9900‐90050). In all three formalin‐fixed samples both the larger (236 bp) and smaller (105 bp) fragments (arrows) were readily amplified, but in Stieve‐ and GR‐fixed samples only the smaller fragment could be amplified (in two of three samples in each case) indicating poorer DNA preservation. No DNA amplification was detected in any of the Bouin‐fixed samples, consistent with Qubit fluorometer measurements, suggesting DNA degradation. (C) Diagrammatic illustration of a testis containing multiple independent mutant clones, the geographical course of clonal expansions within seminiferous tubules (red, yellow) and examples of tubular cross‐sections of immunopositive clones containing pathogenic mutations. Scale bars: 500 μm. See Maher *et al*. (2014) for further discussion.

As reported by Lim *et al*. (2012), in three of the six testes originally studied, we also identified a second phenomenon: at low magnification, we observed cross‐sections of tubules showing an overall darker staining for antibodies to three different spermatogonial antigens (MAGEA4, FGFR3 and phospho‐AKT), involving cells all around the tubular circumference and sometimes extending towards the lumen (Fig. 1A). At higher magnification, it became apparent that the strong staining pattern observed in these ‘immunopositive tubules’ was because of increased density of spermatogonia, which sometimes comprised multiple layers. This unusual pattern was typically observed in groups of two or more adjacent tubular cross‐sections and could be clearly discerned over up to 59 serial 5 μm sections. By reconstructing the three‐dimensional structure of the tubules, we also showed that neighbouring immunopositive tubules were often contiguous, indicating that these immunopositive appearances extended over several millimetres to centimetres of tubular length. We concluded that these immunopositive tubules matched the expected characteristics for selfish spermatogonial selection much more closely than microclones, and expanded on this idea in a review published in this journal (Maher *et al*., 2014). Although Pohl *et al*. (2016) briefly acknowledged our observations of this second phenomenon, it seems they could not find similar features in their own Bouin's‐fixed material. There are three possible explanations for this. First, although they did not quantify the total volume of testes screened in their biopsies, this may have been insufficient as immunopositive tubules are rare, and biopsies yield sparse material compared to whole testis sections (for comparison in our previous study (Lim *et al*. 2012) ~10–30 mm^3^ of each tissue block was analysed). Second, in Bouin's‐fixed material, the appearances may have been more obvious using the additional antibodies (FGFR3, phospho‐AKT) described in Lim *et al*. (2012), but this was not investigated by Pohl *et al*. (2016). Third, compared to formalin fixation, Bouin's fixation may be intrinsically less suitable for immunohistochemical detection of selfish spermatogonial selection. The poor performance of some antibodies in immunohistochemistry of Bouin's‐fixed testicular tissue has been noted previously (Oosterhuis *et al*., 2011). Whatever the correct explanation, a further serious drawback of Bouin's fixation is discussed below.

Because reasonable doubt remained over whether the immunopositive tubules described by Lim *et al*. (2012) really did represent selfish spermatogonial clones, we set out to test this hypothesis directly by isolating and analysing the DNA contained within them. We first extended our immunohistochemical screen to a further 42 FFPE archival tissue blocks sampled from 15 testes (age range 39–96 years), identifying immunopositive tubules in 24 (57%) of them (from 13 of 15 testes). Genetic analysis of this material was experimentally challenging, because the DNA extracted from FFPE tissue is degraded; nevertheless, provided that FFPE material was less than 6 years old, we were able to implement a robust pipeline involving laser capture microdissection, whole genome amplification, and targeted HaloPlex‐based sequencing of >100 genes. An example of the results from one of the testes analysed in our study (Maher *et al*., 2016) is shown in Fig. 1A. Overall, we identified 11 distinct gain‐of‐function mutations in five genes (*FGFR2*,* FGFR3*,* PTPN11*,* HRAS* and *KRAS*) from a majority (16/22) of immunopositive tubules analysed; these genes act in growth factor receptor‐RAS signalling, and all mutations have known associations with severe diseases, ranging from congenital or perinatal lethal disorders, to somatically acquired cancers. These mutations were specific to the immunopositive tubules only, being undetectable in adjacent tubules with normal immunostaining (Fig. 1A). In other words, this work directly and unambiguously links the distinct MAGEA4‐/FGFR3‐/phospho‐AKT immunopositive appearance of tubules with the presence of acquired selfish mutations in these elongated cellular clones. Of note, most such tubules showed variably impaired spermatogenesis when compared to their normal neighbours, hinting that some might be incompatible with the formation of mature spermatozoa (Maher *et al*., 2016).

Whilst we contend that these data (Maher *et al*., 2016) answer the request by Pohl *et al*. (2016) for ‘a more careful interpretation of such spermatogonial clusters…to unequivocally determine “selfish spermatogonial selection”‐manifestations’, we do hope that further research will now be undertaken to explore the cellular characteristics of immunopositive tubules, and their relationship to age, in much greater detail. In this regard, we wish to highlight a serious drawback with the investigation of testes using Bouin's fixative (despite the superior cellular morphology compared to formalin fixation). Unfortunately we have found that the quality of DNA extracted from Bouin's (as well as Stieve and GR, other commonly used fixatives for testicular biopsies) is substantially inferior to FFPE DNA (Fig. 1B), and this result is corroborated by other observations (Ben‐Ezra *et al*., 1991; Gall *et al*., 1993; Benerini Gatta *et al*., 2012). For the field to move beyond the descriptive stage, it will be essential to correlate cellular appearances with molecular analysis, as presented in Fig. 1A and by Maher *et al*. (2016). For example, by applying our protocol to adjacent sections, we have now established that the tubule labelled (C3) in Fig. 2 of Pohl *et al*. (2016) [an image that was copied from Lim *et al*. (2012)], actually contains the *FGFR3* c.1118A>G (p.Y373C) substitution. This corresponds to a known pathogenic mutation, previously described in bladder tumours as well as causative of the germline disorder thanatophoric dysplasia; hence the immunopositive appearance of the tubule (C3) is conclusively not an artefact [the analysis is shown in Fig. S3 of Maher *et al*. (2016)].

We also wish to correct some misunderstandings about mutation frequency in the article by Pohl *et al*. (2016). These authors imply that the identification of immunopositive tubules in three of six randomly assigned testes ‘contradicts the very low mutation prevalence of the related diseases (e.g., achondroplasia, 1/25,000)’ and that there is a ‘discrepancy between the abundance of such clusters which should be very rare and the high amount of affected testes found in the small cohort of men investigated’. We believe that no contradiction exists. In the study by Maher *et al*. (2016), we were able to document three instances, involving different testes, where distinct immunopositive tubules, even within a single section, turned out to harbour different, mutually exclusive, mutations. Hence, we view selfish spermatogonial selection as a prevalent phenomenon, occurring on multiple occasions and involving independent mutations, even within a single testis (illustrated diagrammatically in Fig. 1C). Our conclusions on this point are corroborated by work from the Arnheim group, which has documented, using quantitative DNA analysis, the independent spatial distributions of four different mutations across four of the testes (59089, 374‐1, 374‐2 and 854‐2) they analysed (Qin *et al*., 2007; Choi *et al*., 2008, 2012; Yoon *et al*., 2013). Nevertheless, we expect the absolute risk of transmitting any individual mutation to be low, for three reasons. First, given the estimate that the overall length of seminiferous tubules in a pair of human testes is around 800 m (Glass, 2005), a clone extending along 5 cm of tubule (approximately the maximal length we have been able to document to date) would yield a mutation level of only 1 in 32,000. Second, we have shown a general trend for selfish clones to be associated with impaired spermatogenesis (Maher *et al*., 2016), which is expected to reduce transmission to the next generation. Third, most fathers in the population are young, and will have fewer and smaller clones, because elongation of clones is age‐dependent.

To conclude, we hope that our recent work (Maher *et al*., 2016) will encourage others to explore the extraordinary mutational landscape of the adult human testis. To document the relationship with age will require new, more robustly quantitative methods combining more efficient antibody detection, fixatives that provide superior DNA recovery, whole genome sequencing and (as recently presented for the mouse testis; Nakata *et al*., 2015) computational reconstruction.
